# Supplementary material for: Diagnostic Value of Six Thyroid Imaging Reporting and Data Systems (TIRADS) in Cytologically Equivocal Thyroid Nodules
Source: J Clin Med. 2020 Jul 17;9(7):2281. doi: 10.3390/jcm9072281 (PMC7408998; doi:10.3390/jcm9072281)

Table S1. Comparison of the incidence of US malignancy features and other sonographic features in the nodules with unequivocal (UC) and equivocal (EC) FNA results in relation to the histopathological outcome: benign lesion vs thyroid malignancy.

**Sonographic feature**	**UC (460)**			**EC (540)**								
	**BL (298) MN (162)**			**FLUS/AUS (329)**			**SFN/SHT (167)**			**SM (44)**		
**risk features**	**Ben. (298) No/%**	**Mal. (162) No/%**	**p**	**Ben. (294) No/%**	**Mal. (35) No/%**	**p**	**Ben. (148) No/%**	**Mal. (19) No/%**	**p**	**Ben. (10) No/%**	**Mal. (34) No/%**	**p**
marked hypoechogenicity	7/2.3	58/35.8	<0.0001	20/6.8	7/20.0	<0.01	14/9.5	6/31.6	<0.01	0/0.0	8/23.5	<0.05
hypoechogenicity	140/47.0	144/88.9	<0.0001	201/68.4	29/82.9	NS	111/75.0	15/78.9	NS	6/60.0	29/85.3	NS
solid echostructure	186/62.4	155/95.7	<0.0001	224/76.2	29/82.9	NS	132/89.2	19/100.0	NS	8/80.0	29/85.3	NS
more solid than cystic echostructure	232/77.9	162/100.0	<0.0001	271/92.2	34/97.1	NS	145/98.0	19/100.0	NS	9/90.0	33/97.1	NS
taller than wide	32/10.7	59/36.4	<0.0001	28/9.5	6/17.1	NS	21/14.2	5/26.3	NS	0/0.0	7/20.6	NS
irregular margins	11/3.7	69/42.6	<0.0001	11/3.7	6/17.1	<0.001	6/4.1	1/5.3	NS	0/0.0	16/47.1	<0.05
microcalcifications	4/1.3	39/24.1	<0.0001	8/2.7	7/20.0	<0.0001	9/6.1	2/10.5	NS	0/0.0	5/14.7	NS
macrocalcifications (without micro)	23/7.7	21/13.0	NS	21/7.1	3/8.6	NS	5/3.4	2/10.5	NS	0/0.0	3/8.8	NS
isolated rim calcifications	11/3.7	4/2.5	NS	5/1.7	1/2.9	NS	7/4.7	3/15.8	NS	0/0.0	0/0.0	-
pathological vascularization	32/10.7	29/17.9	<0.05	59/20.1	10/28.6	NS	44/29.7	6/31.6	NS	4/40.0	9/2.6	NS
**other features**												
more (or equally) cystic than solid echostructure	29/9.7	0/0.0	<0.0005	11/3.7	1/2.9	NS	1/0.7	0/0.0	NS	0/0.0	1/2.9	NS
spongiform echostructure	37/12.4	0/0.0	<0.0001	11/3.7	0/0.0	NS	1/0.7	0/0.0	NS	1/10.0	0/0.0	NS

US – ultrasonographic; BL - benign lesion; FLUS/AUS - follicular lesions of undetermined significance/atypia of undetermined significance; SFN/SHT - suspicion of follicular neoplasm/ suspicion of Hürthle cell tumor, SM - suspicion of malignancy MN - malignant neoplasm, Ben. – benign lesion in histopathological outcome, Mal. – thyroid malignancy in histopathological outcome.

Table S2. Data on the diagnostic efficacy of analyzed TIRADSs in the examined groups of nodules - data for the thresholds set one category below the category that gave the highest ACC values.

TIRADS/ guideline	threshold category	SEN	SPC	ACC	PPV	NPV	% of nodules	SEN	SPC	ACC	PPV	NPV	% of nodules
		UC						SM					
3A-T	2	100.0	16.4	45.9	39.4	100.0	89.3	97.1	10.0	77.3	78.6	50.0	95.5
K-T	4	93.8	61.7	73.0	57.1	94.8	57.8	82.4	50.0	75.5	84.8	45.5	75.0
EU-T	4	96.3	54.4	69.1	53.4	96.4	63.5	88.2	50.0	79.5	85.7	55.6	79.5
Kw-T	4b	93.2	62.8	73.5	57.6	94.4	57.0	82.4	50.0	75.0	84.8	45.5	75.0
ACR-T	4	95.1	57.0	70.4	54.6	95.5	61.3	85.3	50.5	77.3	85.3	50.0	77.3
ATA-T	4	93.8	60.7	72.4	56.5	94.8	58.5	82.4	50.0	75.0	84.8	45.5	77.3
		FLUS/AUS						SFN/SHT					
3A-T	3	100.0	3.7	14.0	11.0	100.0	96.7	100.0	0.7	12.0	11.4	100.0	99.4
K-T	5	82.9	41.2	45.6	14.4	95.3	61.4	84.2	24.3	31.1	12.5	92.3	76.6
EU-T	5	88.6	29.9	36.2	13.1	95.7	72.0	84.2	20.3	27.5	11.9	90.9	80.2
Kw-T	4c	80.0	41.8	45.9	14.1	94.6	60.5	84.2	24.3	31.1	12.5	92.3	76.6
ACR-T	5	82.9	37.1	41.9	13.6	94.8	65.0	84.2	21.6	28.7	12.1	91.4	82.6
ATA-T	5	82.9	40.8	45.3	14.3	95.2	61.7	84.2	24.3	31.1	12.5	92.3	76.6

Figure S1. Selection of nodules for the study

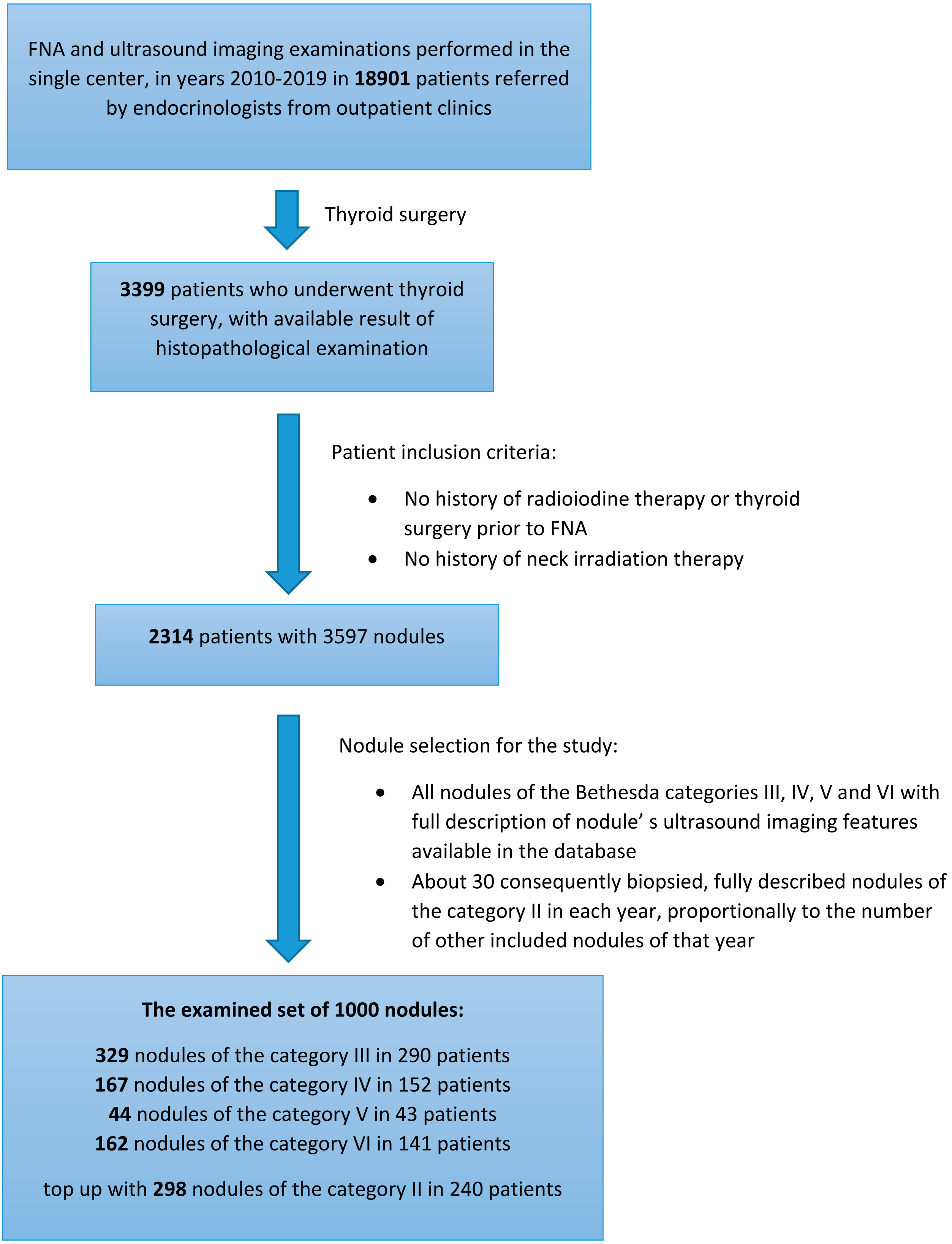

Supplement: Supplementary file 1 [file jcm-09-02281-s001.pdf]
